# Host-associated coral reef microbes respond to the cumulative pressures of ocean warming and ocean acidification

**DOI:** 10.1038/srep19324

**Published:** 2016-01-13

**Authors:** N. S. Webster, A. P. Negri, E. S. Botté, P. W. Laffy, F. Flores, S. Noonan, C. Schmidt, S. Uthicke

**Affiliations:** 1 Australian Institute of Marine Science, Townsville Qld Australia

## Abstract

Key calcifying reef taxa are currently threatened by thermal stress associated with elevated sea surface temperatures (SST) and reduced calcification linked to ocean acidification (OA). Here we undertook an 8 week experimental exposure to near-future climate change conditions and explored the microbiome response of the corals *Acropora millepora* and *Seriatopora hystrix*, the crustose coralline algae *Hydrolithon onkodes*, the foraminifera *Marginopora vertebralis* and *Heterostegina depressa* and the sea urchin *Echinometra* sp. Microbial communities of all taxa were tolerant of elevated *p*CO_2_/reduced pH, exhibiting stable microbial communities between pH 8.1 (*p*CO_2_ 479–499 μatm) and pH 7.9 (*p*CO_2_ 738–835 μatm). In contrast, microbial communities of the CCA and foraminifera were sensitive to elevated seawater temperature, with a significant microbial shift involving loss of specific taxa and appearance of novel microbial groups occurring between 28 and 31 °C. An interactive effect between stressors was also identified, with distinct communities developing under different *p*CO_2_ conditions only evident at 31 °C. Microbiome analysis of key calcifying coral reef species under near-future climate conditions highlights the importance of assessing impacts from both increased SST and OA, as combinations of these global stressors can amplify microbial shifts which may have concomitant impacts for coral reef structure and function.

Rapidly rising atmospheric carbon dioxide (CO_2_) is causing ocean acidification (OA) and an increase in sea surface temperatures (SST)^1^. The partial pressure of CO_2_ in the ocean (*p*CO_2_) is projected to increase to around 1000 ppm by 2100 with associated declines in oceanic pH and carbonate availability^2^, while sea surface temperatures are set to increase by 1–3 °C over the same period^1^. Mass coral bleaching and mortality events are associated with elevated SST^3^ and it is anticipated that some Great Barrier Reef (GBR) coral species will exceed their thermal thresholds within the next century under IPCC projected climate scenarios^4^. In addition, increasing *p*CO_2_ reduces calcification in reef-building species like corals and crustose coralline algae (CCA)^5^,^6^,^7^ and combined with the impacts of elevated SST, acidification poses a long-term threat to the viability of coral reefs globally^4^. The impact of these cumulative pressures was recently highlighted in a meta-analysis of climate change research indicating that a combination of climate-related stressors can have greater deleterious effects on marine organisms than stressors applied in isolation^8^. However, to date, relatively few coral reef studies have examined the interactive effects of temperature and OA and even fewer have explored the responses of the host-associated symbiont communities.

Reef invertebrates host a diversity of symbiotic microbes, many of which are vital for the continued health of the animal hosts. For example, coral-associated microbes undertake essential functions including fixation and passage of nitrogen and carbon to the host^9^,^10^,^11^, metabolism of DMS and DMSP^12^,^13^ and the production of secondary metabolites and antimicrobial compounds to defend their hosts against predation and pathogenic microorganisms^14^,^15^,^16^. Microbes associated with CCA have also been shown to induce settlement and metamorphosis of coral reef larvae^17^,^18^,^19^. Considering this wide range of symbiotic and ecologically important functions, environmental conditions which disrupt the composition or abundance of marine microbes could have significant effects on host fitness and survival as well as overall reef ecosystem health. Despite this, our understanding of how host-associated microbes respond to elevated SST and OA is limited and we have no knowledge of how they respond to the interactive effects of these climate stressors^20^,^21^.

Compositional and functional changes in invertebrate microbiomes in response to elevated SST have been reported from a range of reef taxa^22^,^23^,^24^,^25^, including recent evidence that thermal stress can reduce expression of functions that mediate symbiotic host-microbial partnerships via disruption to nutritional interdependence and molecular interactions^26^. Fewer studies have investigated the effects of OA on marine invertebrate-associated bacteria, despite the fact that increases in CO_2_ or reductions in pH can have consequences for microbially-driven nutrient cycling^27^, nitrification^28^ and iron availability^29^. In the few studies which have experimentally explored the effects of OA on marine microbial partnerships, a reduction in pH from 8.1 to 7.9 was found to alter the microbial community in coral, crustose coralline algae (CCA), foraminifera and reef biofilms^30^,^31^. In addition, *in situ* ocean acidification studies at CO_2_ seeps have documented changes in coral microbial communities at the extreme pH of 7.3^32^ and in the microbial communities of two coral and two sponge species at a CO_2_ vent where carbonate chemistry varied between pH 8.01-7.28^33^.

To adequately predict climate change impacts on reef invertebrates we need to enhance our understanding of the sensitivity of host-associated microbial assemblages to near future climate scenarios. In this study we used next generation sequencing (NGS) to explore the interactive effects of elevated SST and OA on the microbial communities inhabiting 4 key calcifying reef phyla (corals, foraminifera, echinoderms and crustose coralline algae) following an eight week exposure in an experimental system.

## Results

### Host Health

No visible signs of compromised health were evident in any of the host taxa over the experimental duration. As sub-lethal stress in the host was not determined, it is not possible to deconstruct whether observed microbial shifts are a direct result of host stress or host selection of different microbial phylotypes under different treatment conditions.

### Overall community composition

**Figure 1:**
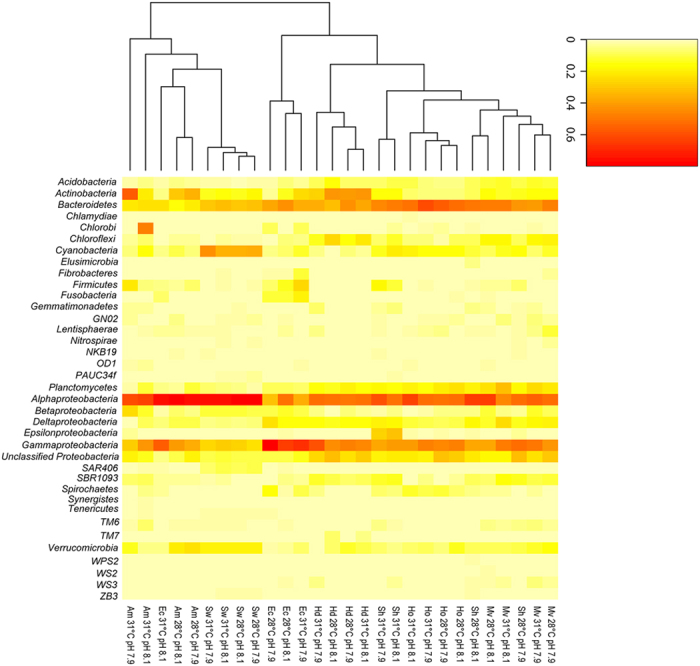
Heatmap illustrating the distribution of 454 amplicon reads for each species across all temperature and *p*CO_2_ treatments. The average number of reads per taxa is calculated from the percentage of total reads in each sample. The heatmap represents the Phyla level with the exception of the *Proteobacteria* which have been represented at the Class level. Am = *Acropora millepora,* Sh = *Seriatopora hystrix*, Ho = *Hydrolithon onkodes*, Hd = *Heterstegina depressa*, Mv = *Marginopora vertebralis*, Ec = *Echinometra* sp. and Sw = seawater. The mean percentage ± standard error of each bacterial taxa in individual samples is presented in SOM Tables 1–7.

Averaged microbial community composition at the phyla/class level clustered primarily according to host species (Fig. 1). The only grouping according to pH/temperature treatment was a separation of the *S. hystrix* samples based on an elevated relative abundance of sequences related to the Class *Epsilonproteobacteria* at 31 °C (Fig. 1). The microbial community of the foraminifera *M. vertebralis* was more similar to the community of the coral *S. hystrix* than to the community of the other foraminifera *H. depressa*, largely due to a higher proportion of sequences related to *Deltaproteobacteria* and a lower proportion of sequences related to *Actinobacteria*. However, overall, both foraminifera species and *S. hystrix* were dominated by sequences related to *Alphaproteobacteria, Gammaproteobacteria* and *Bacteroidetes* but also hosted abundant sequences within the phyla *Chloroflexi, Cyanobacteria* and *Planctomycetes*. The phyla/class level microbial composition in the CCA *H. onkodes* was dominated by sequences related to *Alpha* and *Gammaproteobacteria*, *Bacteroidetes*, *Cyanobacteria* and *Planctomycetes* (Fig. 1). The sea urchin *Echinometra* sp. in the 31 °C/pH 8.1 treatments had a microbial community that was more similar to the microbial community of *A. millepora* than the *Echinometra* sp. samples at the other treatment conditions, largely due to an elevated relative abundance of sequences related to *Alphaproteobacteria*. Overall, *Echinometra* sp. was dominated by sequences affiliated to *Alphaproteobacteria, Gammaproteobacteria* and *Bacteroidetes* but also hosted abundant sequences related to *Deltaproteobacteria, Firmicutes* and *Fusobacteria.* The coral *A. millepora* had a more variable microbial community including abundant representatives of *Alpha* and *Gammaproteobacteria, Actinobacteria* and *Bacteroidetes* and samples at 31 °C/pH 8.1 also contained an abundance of sequences related to *Chlorobi*. A relatively high proportion of unclassified sequences within the *Proteobacteria* was also evident across all invertebrate species. Seawater samples from all treatments were highly similar, being dominated by sequences related to *Alphaproteobacteria*, *Cyanobacteria*, *Bacteroidetes* and a low abundance of sequences related to *Gammaproteobacteria* and *Verrucomicrobia.*

### Temperature and pH impacts

Between treatment comparisons of host associated microbiomes were performed independently for each species. No analyses are made regarding between species differences as species-specific responses are likely to be affected by temporal factors. The composition of host-associated microbial communities was unaffected by pH/*p*CO_2_ alone for all species.

However, despite the overall microbial community composition in the coral *A. millepora* not differing between *p*CO_2_ treatments, it is noteworthy that the known *p*CO_2_ sensitive coral symbiont *Endoziocomonas* sp. (OTU 3) is significantly reduced at low pH (t(8) = −2.577, p = 0.0328) with this symbiont comprising an average of 5.1 ± 2.9% of the sequence reads at pH 8.1 and only 0.9 ± 0.2% of the total reads in *A. millepora* at pH 7.9.

**Figure 2:**
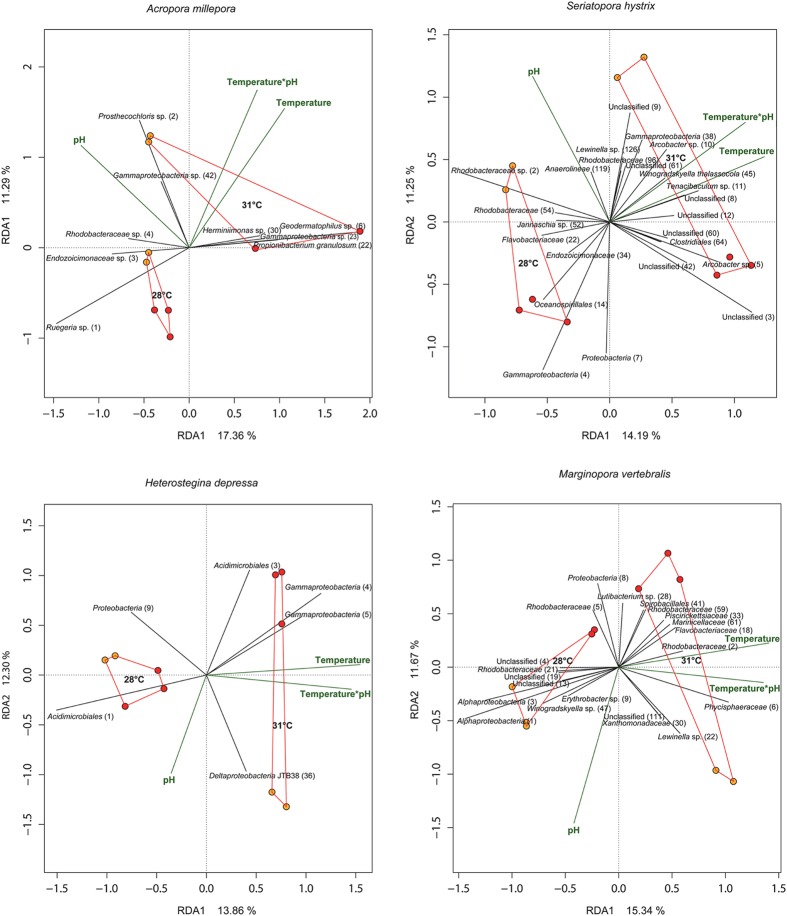
Distance based redundancy analyses (dbRDA) using a Bray Curtis distance matrix to summarise the variation in composition of bacterial communities for *A. millepora*, *S. hystrix*, *H. depressa* and *M. vertebralis* that was attributable to treatment (ie temperature or *p*CO_2_). pH 7.9 is represented by red circles and pH 8.1 by orange circles and all points within each temperature are joined. For clarity, vectors and taxonomic affiliations (including OTU identifiers) are only shown for the 1% most discriminating OTUs.

**Figure 3:**
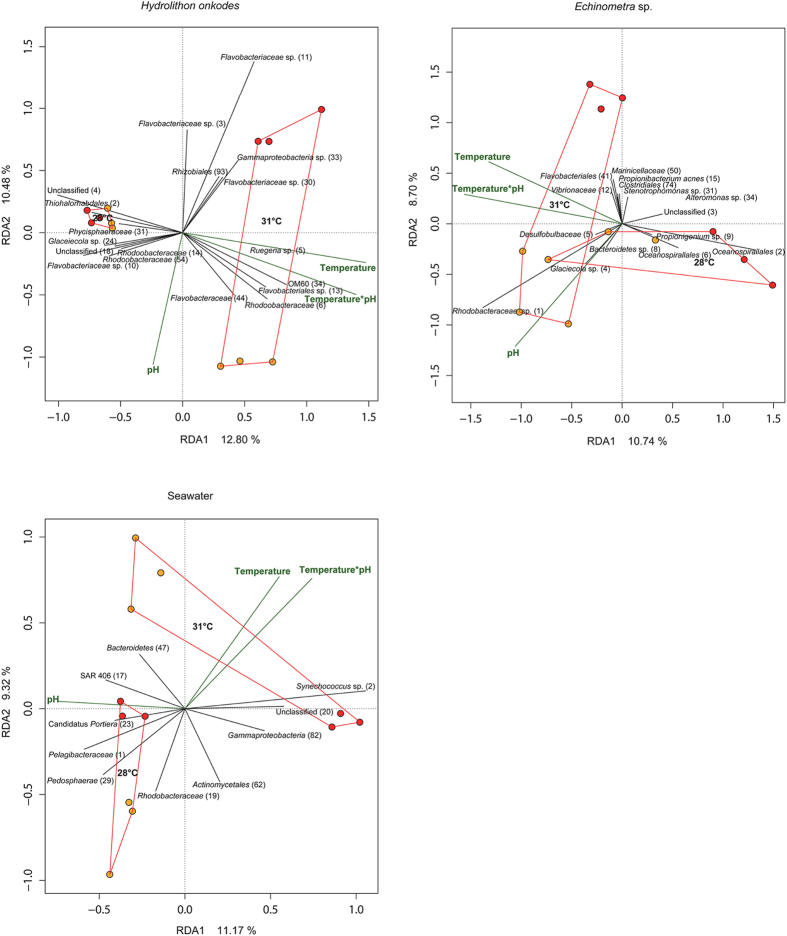
Distance based redundancy analyses (dbRDA) using a Bray Curtis distance matrix to summarise the variation in composition of bacterial communities for *H. onkodes*, *Echinometra* sp. and seawater that was attributable to treatment (ie temperature or *p*CO_2_). pH 7.9 is represented by red circles and pH 8.1 by orange circles and all points within each temperature are joined. For clarity, vectors and taxonomic affiliations (including OTU identifiers) are only shown for the 1% most discriminating OTUs.

The composition of host-associated microbial communities was unaffected by temperature in both coral species and *Echinometra* sp. but differed significantly between 28 °C and 31 °C in the CCA and both foraminifera species (Figs 2 and 3, Table 1). An interactive effect between stressors was also evident with *p*CO_2_ causing more divergent community compositions at the higher seawater temperature (Figs 2 and 3, Table 1). Although the interactive effect was not significant in either coral species, the trend of a larger *p*CO_2_ effect at higher temperature was still observed in both *A. millepora* and *S. hystrix* and a significant interactive effect was evident in the seawater.

The primary drivers of temperature-related differences in the microbial community of the CCA *H. onkodes* was an increase in OTUs affiliated with *Flavobacteriales* (OTU 13), *Ruegeria* sp. (OTU 5) and OM60 (OTU 34) at higher temperature and a decreased relative abundance of OTUs affiliated with *Phycisphaeraceae* (OTU 31), *Thiohalorhabdales* (OTU 2) and an unclassified bacterium (OTU 4) (Fig. 3). There was also a strong correlation between the *Flavobacteriaceae* OTU 11 and increasing *p*CO_2_. In both foraminifera species the biggest driver of temperature variation was the loss of OTUs at 31 °C (Fig. 2). For example, in *H. depressa* the significant difference in communities between temperature treatments was largely due to the loss of a single *Acidomicrobiales* (OTU 1) at 31 °C which represented a dominant part of the community in this species at 28 °C. In contrast, *H. depressa* hosted a dramatic increase in another *Acidomicrobiales* (OTU 3) at elevated *p*CO_2_. In *M. vertebralis*, OTUs related to *Alphaproteobacteria* (OTUs 1, 3) and *Rhodobacteriaceae* (OTU 21) as well as 3 unclassified OTUs (OTUs 4, 13, 19) were negatively correlated with 31 °C and a *Rhodobacteriaceae* (OTU 2) and a *Phycisphaeraceae* (OTU 6) became more abundant at the higher temperature. A significant interactive effect of temperature and *p*CO_2_ was observed in the seawater microbial community and this was primarily driven by a much higher abundance of the *Synechococcus* OTU 2 at elevated *p*CO_2_ (Fig. 3, Table 1).

## Discussion

Here we show a significant interacting effect between pH and seawater temperature on the composition of the microbial communities inhabiting the CCA *H. onkodes* and the foraminfera *M. vertebralis* and *H. depressa,* with *p*CO_2_ having a greater influence at the elevated temperature. Interestingly, at ambient temperature (28 °C) the microbial communities of all coral, CCA, foraminifera and echinoderm species were stable between pH 8.15 (*p*CO_2_ 479–499 μatm) and pH 7.96 (*p*CO_2_ 738–835 μatm), although it is possible that longer term incubations may have revealed additional patterns in microbial community dynamics for these host taxa. Previous research has documented bacterial community shifts in some of these taxa between pH 8.10 (*p*CO_2_ = 464 μatm) and pH 7.90 (*p*CO_2_ = 822 μatm)^30^,^31^. There are multiple factors which result in apparent differences in community responses between studies, including different: host species; experimental conditions; exposure durations; microbial compositions of the seawater; acclimation periods and analytical approaches. This experimental variability confounds direct comparisons between studies and underscores the need for caution when inferring changes to broader phyla levels or community patterns. For instance, all previous aquarium-based studies have employed microbial profiling or clone sequencing approaches to describe the changes in host-associated microbial communities, resulting in more restricted sequencing depth and much lower resolution than was achieved in the present study utilising a next generation sequencing (NGS) approach. The slightly lower *p*CO_2_ employed in the present study may also have contributed to the microbial stability we observed across *p*CO_2_ treatments at ambient temperature when compared to previous research. It is also important to note that, while aquarium-based experiments with static environmental conditions (temperature, light, pH etc) do not accurately reflect *in situ* field conditions, that this approach is advantageous for between-study stress threshold comparisons and therefore represents a valid approach for investigating the potential sensitivity of host associated microbiomes to climate change.

Consequences of hosting stable vs sensitive microbial communities under OA likely vary between species. Many studies have documented a decline in host health associated with the loss of key symbionts that perform essential functions for the host^26^,^32^,^34^,^35^,^36^. For example, in a recent study of corals inhabiting a naturally occurring CO_2_ seep in Papua New Guinea, a loss of functionally important *Endozoicomonas* symbionts in *A. millepora* at the CO_2_ seep was linked to a significant reduction in the abundance of this coral species at high *p*CO_2_^33^. In the current study, an *Endozoicomonas* OTU was also found to be significantly reduced at pH 7.9, confirming the apparent pH sensitivity of this important coral symbiont. *Endozoicomonas* have been shown to live endosymbiotically within coral endoderm^37^, have the ability to metabolize DMSP^13^ and are thought to play a role in nutrient acquisition and cycling of organic compounds^33^. The loss of this symbiont is therefore expected to have deleterious effects on host health, although this was not visibly evident over the short experimental period in the current study. However, it is also important to note that flexibility in symbiosis has also been suggested as a mechanism that may expand the niche breadth of some invertebrate species under climate change when microbial shifts facilitate greater nutritional benefit, enhanced scope for growth or other competitive advantages^33^.

In contrast to the overall stability of microbial communities to near future *p*CO_2_ conditions, the communities of the CCA and foraminifera species were found to be highly sensitive to elevated SST (ambient +3 °C). The CCA *Neogoniolithon fosliei* has previously been shown to exhibit high sensitivity to elevated SST, with bleaching and a reduction in maximum quantum yield (*F*_*v*_*/F*_*m*_) at 31 °C and a large shift in microbial community structure at 32 °C with a concomitant 50% reduction in the ability of the CCA to induce coral larval metamorphosis^25^. The microbial shift at 32 °C in *Neogoniolithon fosliei* involved an overall increase in *Bacteroidetes* and a reduction in *Alphaproteobacteria.* Whilst this broad phylogenetic effect was not evident in *H. onkodes* at elevated temperature, we did detect an increase in the *Bacteroidetes* OTU 13 and a decrease in the *Alphaproteobacteria* OTUs 14 and 54 in *H. onkodes* at 31 °C. Importantly, the impact of elevated seawater temperature was exacerbated by the combination of reduced pH/elevated *p*CO_2_ indicating that the simultaneous pressures magnify effects on the microbial community in comparison with temperature alone.

This is the first study to investigate the potential impacts of rising SST on the microbial communities of foraminifera, which is surprising as foraminifera are reported to host the highest microbial diversity of all coral reef invertebrate taxa investigated^38^ and both the host and symbiont physiologies are sensitive to increasing SST^39^,^40^,^41^,^42^ with the dominant *Symbiodinium* clade in *M. vertebralis* being largely determined by SST^43^. This study clearly demonstrates the high thermal sensitivity of foraminifera-associated microbial communities, with the significant microbial shifts primarily attributed to the loss of specific bacterial taxa at 31 °C and the impact being greater under reduced pH/elevated *p*CO_2_ conditions. Considering the ecological importance of benthic foraminifera as generators of reef carbonate^44^, future studies should investigate the implications of these microbial shifts for host health.

The stability of the *A. millepora*-associated microbial community between temperature treatments is consistent with previous observations that bacterial community profiles of *A. millepora* on the GBR are maintained throughout the year despite seasonal variation in temperature and other environmental parameters^45^. However, this may be a species-specific response as other coral species have been shown to host highly dynamic microbial communities across seasons, bleaching events and under experimentally elevated seawater temperatures^22^,^46^,^47^,^48^. Consistent with the known thermal sensitivity of *S. hystrix*^49^, the microbial community response to temperature in this species was only just outside the range of significance (p = 0.058).

Whilst neither temperature nor *p*CO_2_ alone had a significant effect on the seawater microbial community, a significant interactive effect was observed and primarily driven by a much higher abundance of photosynthetic *Synechococcus* at elevated *p*CO_2_ at higher temperature. Planktonic *Synechococcus* have previously been shown to increase carbon fixation at elevated *p*CO_2_, although this effect is short term with acclimation of cellular physiology generally occurring within 24–72h^50^. An elevated abundance of symbiotic *Synechococcus* under conditions of ocean acidification has also recently reported for sponges at a naturally occurring CO_2_ seep^33^, although the increase in planktonic *Synechococcus* within aquarium seawater did not appear to influence the composition of host-associated microbial communities in any taxa within the present study.

## Summary

Deep microbial sequencing of samples experimentally exposed to climate change conditions predicted for 2100 has revealed that bacterial communities of many key calcifying coral reef taxa are capable of withstanding short-term exposure to elevated *p*CO_2_/reduced pH. In contrast, the microbial communities of CCA and foraminifera were sensitive to elevated seawater temperature, undergoing significant shifts between 28 and 31 °C which were exacerbated under elevated *p*CO_2_/reduced pH. Although further studies are required to ascertain the consequences of these microbial shifts for host health and fitness, the thermal sensitivity of microbial taxa associated with CCA and foraminifera highlights the need for a holobiont approach for environmentally relevant assessments of invertebrate vulnerability to climate change.

## Methods

All specimens for the experiment were collected in September 2011 and transported to the aquarium system at the Australian Institute of Marine Science (AIMS). Colonies of the two coral species *Acropora millepora* and *Seriatopora hystrix*, the CCA *Hydrolithon onkodes* and individuals of the sea urchin *Echinometra* sp. A (genetically congruent with the species ‘A’ complex defined in^51^ and hereafter called *Echinometra* sp.) were collected from Davies Reef (18°50.558’S, 147°37.618’E) at a depth of 2–5 m. Specimens of the foraminifera *Marginopora vertebralis* were collected at 1 m depth and *Heterostegina depressa* at 8–12 m depth at Orpheus Island in the central GBR (18° 34.133′ S, 146° 28.917 E). Small coral branches were fragmented from the colonies, strung in the aquaria with fine nylon fishing line and allowed to heal for 3 weeks prior to transfer to experimental tanks. Circular cores of CCA (10 mm^2^) were mounted onto individual recessed PVC slides with superglue so that only the live CCA was exposed to seawater. The mounted CCA was allowed to acclimate in the indoor aquarium for 3 weeks prior to treatment, a sufficient period for new growth to become apparent and seal over bare skeleton. Foraminifera were cultured in flow-through six-well-plates (Nunc, Denmark) which were placed into each aquarium and covered with plankton mesh to prevent specimens from escaping. To avoid interaction effects between motile *Echinometra* sp., and other organisms, the sea urchins were maintained in separate aquaria supplied by the same water using an identical experimental setup.

### Experimental Design

In order to identify changes in microbial community composition due to different OA and temperature conditions projected for 2100^1^, the experiment was conducted in a flow-through aquarium system using a 2 × 2 factor design with all other physical, chemical and biotic factors being held constant. Corals, foraminifera, sea urchins and CCA (n = 12) were deployed into triplicate aquaria for each pH/temperature treatment. Seawater treatments were set at pH_NIST_ 8.10 or 7.90 using a computer controlled CO_2_ aquarium dosing facility described in^52^. Briefly, seawater was 1 μm filtered then pH controlled in 4 × 450 l header tanks (two header tanks per treatment pH) using a CO_2_ gas injection system (AquaMedic, Germany). This system facilitated identical water chemistry from source header tanks but no other connectivity between replicate treatment tanks. The system was regulated by feedback every 30 s from NIST calibrated potentiometric sensors (±0.01 pH unit) in a treatment tank. The seawater temperatures for each pH treatment were adjusted to either 28 or 31 °C using titanium heating rods in the header tanks which were monitored with a computer-controlled data logger system (CR 1000, Campbell Scientific, Australia) that incorporated a logger in a single tank per temperature treatment. All samples were exposed to the two seawater *p*CO_2_ conditions at each temperature for 8 weeks. All 17.5 l aquaria were supplied with 1 μm filtered seawater at a flow rate sufficient to enable 100% turnover every 45 min. Tanks were maintained under a 12:12 h diel light cycle at 180–200 μmol photons m^−2 ^s^−1^ using 55 W, 10000 K compact fluorescent tubes. However, foraminifera specimens were shaded further and received only 10–17 or 38–45 μmol photons m^−2 ^s^−1^ light for *H. depressa* and *M. vertebralis* respectively to account for species-specific light requirements.

Daily pH readings were taken in each treatment tank using a potentiometric pH probe (console: OAKTON, USA; pH probe: EUTECH, USA). In addition, a 250 ml seawater sample was collected from each replicate aquaria and analysed for total alkalinity (A_T_) and dissolved inorganic carbon (C_I_) (Table 2) with these values being determined as previously described (Uthicke *et al.* 2012a). *p*CO_2_, bicarbonate, carbonate and aragonite saturation state (Ω) were derived from pH and TA measurements using CO_2_ SYS^53^.

### DNA Extraction and Sequence Analysis

Prior to DNA extraction, coral tissue was blasted from the skeleton of each nubbin using sterile tips and pressurized air, live CCA tissue was scraped from the surface of the coralline chips using sterile razors, whole individual foraminifera were ground with a sterile mortar and pestle and sea urchin spines were removed with sterile forceps and immediately rinsed in sterile MQ-H_2_O. In addition, seawater (1 l) from each tank was filtered through 0.2 μm sterivex filters. DNA was extracted from all samples using the Power Plant DNA Isolation kit, MoBio Laboratories (Carlsbad, CA) according to the manufacturer’s protocol. Extracted DNA was subsequently quantified using a NanoDrop 2000 UV-V is spectrophotometer (Wilmington, DE, USA), PCR amplified with universal bacterial primers targeting the hypervariable region V1-V3 of the 16S ribosomal rRNA gene with 28F (5′GAGTTTGATCNTGGCTCAG) and 519R (5′GTNTTACNGCGGCKGCTG) and sequenced at the Molecular Research Laboratories (Shallowater, TX, USA) as previously described^33^.

### Sequence analysis

Raw .sff sequence reads from all samples were denoised and processed through the MOTHUR software package (www.mothur.org) with analysis as per the 454 standard operating procedure^54^. Briefly, sequences of length <200 bp, ambiguous base calls, or homopolymer runs exceeding 8 bp were removed. Sequences were aligned with the SILVA 16S rRNA alignment^55^ as a reference and operational taxonomic units (OTUs) were defined using Pre.cluster after removal of singleton sequences, clustering at 3% divergence (97% similarity). Chimeric artefacts were also removed using UCHIME^56^. OTUs were taxonomically classified using a BLAST-based method against the May 2013 curated Greengenes database^57^,^58^ (http://greengenes.secondgenome.com/) and compiled at each taxonomic level into a counts file. OTU classifications were performed separately for each species dataset. Any sequences that were classified as Mitochondria, Eukaryotic or Chloroplast as well as any sequences of unknown origin were filtered out of the dataset. Sequences without hits were rechecked against the NCBI-nr database (http://www.ncbi.nlm.nih.gov/) for 16S rRNA gene sequences retrieved from uncultured bacteria using a BLAST search^57^ with the E-value threshold (0.001). Four sample replicates (*H. depressa* 28 °C/pH 8.1, *H. depressa* 31 °C/pH 8.1, *M. vertebralis* 28 °C/pH 7.9 and *A. millepora* 31 °C/pH 8.1) failed sequencing. Data from each species were analysed separately and each rarefied to account for 16S rRNA gene copy number bias. The raw pyrosequencing reads were submitted to the NCBI Sequence Read Archive under accession numbers SAMN03941220 - SAMN03941247.

### Statistical analysis

Distance-based Redundancy analysis (RDA), also known as constrained analysis of principal coordinates, was used to investigate variation in the bacterial community between treatments for each species independently using a Bray-Curtis distance metric. In contrast to traditional unconstrained principal coordinates analysis, an advantage of RDA is that it enables the inclusion of environmental factors and the testing of their interaction using non-Euclidean distance matrices^59^. As RDA always results in at least some separation of groups in the plots, visual interpretation of the separation is restricted to determination of bacterial OTUs that are associated with each of the environmental factors. The amount of variance explained by individual and combined factors was tested using Monte Carlo permutation tests (999 permutations) in R version 2.12.0. Two RDAs were conducted for each taxon, the first with both seawater temperature and *p*CO_2_ as independent environmental variables and the second allowing an interaction term between them. The distance of each OTU from the origin is proportional to its variance along an axis and its angle relative to the axes reflects its correlation with those axes. For clarity, vectors and taxonomic affiliations are only shown for the most discriminating OTUs (top 1%). Differences in the proportions of sequence reads corresponding to OTU 3 (Endozoicomonas) between pH treatments were compared using t-tests following arcsine-suare root transformation.

## Additional Information

**How to cite this article**: Webster, N. S. *et al.* Host-associated coral reef microbes respond to the cumulative pressures of ocean warming and ocean acidification. *Sci. Rep.* **6**, 19324; doi: 10.1038/srep19324 (2016).

## Supplementary Material

Supplementary Information

## Figures and Tables

**Table 1:** Results of permutation analyses testing the significance and variation explained by temperature and pH for each host species.

Species	Total	Temperature	pH	Total Interaction
*A. millepora*	p = 0.227 (25.9)	p = 0.3208 (12.9)	p = 0.2957 (13.0)	p = 0.4892 (37.8)
*S. hystrix*	p = 0.0624 (24.4)	p = 0.0584 (12.8)	p = 0.3186 (11.6)	p = 0.1645 (34.8)
*H. onkodes*	**p** = **0.0014 (22.0)**	p = **0.0007** (11.8)	p = 0.0669 (10.2)	**p** = **0.0038 (30.8)**
*H. depressa*	**p** = **0.0545 (24.6)**	p = **0.0135** (13.7)	p = 0.5415 (10.9)	**p** = **0.0521 (35.9)**
*M. vertebralis*	**p** = **0.0071 (26.4)**	p = **0.0098** (14.5)	p = 0.2441 (11.9)	**p** = **0.0014 (38.1)**
*Echinometra* sp.	p = 0.4149 (19.4)	p = 0.2630 (9.8)	p = 0.3132 (9.6)	p = 0.5313 (26.9)
Seawater	p = 0.05 (19.6)	p = 0.092 (9.9)	p = 0.1680 (9.7)	**p** = **0.023 (29.2)**

Two models were run for each species, the first to test main factors only and the second to include an interaction term. Numbers in brackets indicate the % of variation explained by each treatment factor and significant results are bold typeset.

**Table 2:** Average water chemistry in treatment tanks containing corals, CCA and foraminifera.

Treatment	pH [NIST]	Temp [°C]	A_T_[μmol/kg SW]	DIC [μmol/kg SW]	*p*CO_2_[μatm]	Ω_Ca_	Ω_Ar_
8.1/28 °C	8.15 (0.05)	28.1 (0.2)	2332 (24)	2031 (10)	479 (38)	5.1 (0.3)	3.4 (0.2)
8.1/31 °C	8.14 (0.05)	30.8 (0.3)	2338 (20)	2025 (6)	499 (32)	5.4 (0.3)	3.6 (0.2)
7.9/28 °C	7.98 (0.05)	27.9 (0.3)	2335 (22)	2134 (19)	738 (65)	3.8 (0.2)	2.5 (0.1)
7.9/31 °C	7.96 (0.03)	30.8 (0.4)	2337 (22)	2142 (16)	835 (85)	3.8 (0.3)	2.6 (0.2)

Values in brackets represent standard deviations for N = 3 sampling periods. pH (NIST) represents the pH value as calculated from measured Alkalinity (A_T_) and values of dissolved inorganic carbon (C_I_).
