# Supplementary material for: Host-associated coral reef microbes respond to the cumulative pressures of ocean warming and ocean acidification
Source: Sci Rep. 2016 Jan 13;6:19324. doi: 10.1038/srep19324 (PMC4725835; doi:10.1038/srep19324)
Supplement: Supplementary Information [file srep19324-s1.doc]

**Supporting Online Material**

**Host-associated coral reef microbes respond to the cumulative pressures of ocean warming and ocean acidification**

Webster, N.S.*, Negri, A.P., Botté, E.S., Laffy, P.W., Flores, F., Noonan, S., Schmidt, C. and Uthicke, S.

Australian Institute of Marine Science, Townsville Qld Australia

*Corresponding Author:

Australian Institute of Marine Science

PMB 3, Townsville Mail Centre, Qld, Australia 4810

E-mail: [n.webster@aims.gov.au](mailto:n.webster@aims.gov.au).

Phone: (+61) 747534151

Fax: (+61) 747725852

Running Title: Microbial responses to elevated temperature and *p*CO_2_

| **Bacterial Taxa** | **Am 28°C pH 8.1**  **Mean ± SE** | | **Am 28°C pH 7.9**  **Mean ± SE** | | **Am 31°C pH 8.1**  **Mean ± SE** | | **Am 31°C pH 7.9**  **Mean ± SE** | |
| --- | --- | --- | --- | --- | --- | --- | --- | --- |
| *Acidobacteria* | 0.24 | 0.16 | 0.13 | 0.10 | 0.04 | 0.04 | 0.20 | 0.20 |
| *Actinobacteria* | 8.11 | 4.55 | 11.37 | 6.17 | 31.25 | 28.64 | 3.88 | 1.27 |
| *Bacteroidetes* | 2.37 | 1.19 | 4.06 | 0.76 | 4.87 | 4.00 | 5.26 | 5.02 |
| *Chlamydiae* | 0 |  | 0 |  | 0 |  | 0 |  |
| *Chlorobi* | 0 |  | 0 |  | 0 |  | 20.97 | 20.97 |
| *Chloroflexi* | 0.12 | 0.12 | 0.08 | 0.08 | 0.08 | 0.08 | 0.55 | 0.55 |
| *Cyanobacteria* | 1.03 | 0.55 | 0.29 | 0.16 | 0.40 | 0.40 | 2.25 | 0.75 |
| *Elusimicrobia* | 0 |  | 0 |  | 0 |  | 0 |  |
| *Fibrobacteres* | 0 |  | 0 |  | 0 |  | 0 |  |
| *Firmicutes* | 0.12 | 0.04 | 0.45 | 0.23 | 4.63 | 2.33 | 0.28 | 0.20 |
| *Fusobacteria* | 0 |  | 0 |  | 0 |  | 0 |  |
| *Gemmatimonadetes* | 0 |  | 0 |  | 0.12 | 0.12 | 0.08 | 0.08 |
| GN02 | 0.24 | 0.24 | 0 |  | 0.08 | 0.08 | 0 | 0 |
| *Lentisphaerae* | 0.08 | 0.08 | 0.05 | 0.03 | 0 |  | 0.04 | 0.04 |
| *Nitrospirae* | 0 |  | 0 |  | 0 |  | 0 |  |
| NKB19 | 0.04 | 0.04 | 0 |  | 0 |  | 0 |  |
| OD1 | 0 |  | 0 |  | 0.04 | 0.04 | 0.08 | 0.08 |
| PAUC34f | 0 |  | 0 |  | 0 |  | 0 |  |
| *Planctomycetes* | 0.08 | 0.08 | 1.24 | 0.47 | 0.04 | 0.04 | 1.15 | 0.44 |
| *Alphaproteobacteria* | 60.28 | 5.06 | 54.75 | 4.33 | 35.32 | 35.32 | 37.06 | 1.07 |
| *Betaproteobacteria* | 0.47 | 0.16 | 0.16 | 0.12 | 5.62 | 4.98 | 1.54 | 1.54 |
| *Deltaproteobacteria* | 0.87 | 0.87 | 0.98 | 0.60 | 0 |  | 0.67 | 0.44 |
| *Epsilonproteobacteria* | 0 |  | 0 |  | 0.12 | 0.12 | 0 |  |
| *Gammaproteobacteria* | 15.47 | 7.48 | 12.32 | 3.28 | 7.59 | 4.35 | 16.14 | 6.80 |
| Unclass. *Proteobacteria* | 1.07 | 0.75 | 3.53 | 2.75 | 3.40 | 3.09 | 1.86 | 0.44 |
| SAR406 | 0 |  | 0 |  | 0 |  | 0.04 |  |
| SBR1093 | 0.08 | 0.08 | 0.08 | 0.08 | 0.44 | 0.44 | 0.67 | 0.51 |
| *Spirochaetes* | 0 |  | 0 |  | 0 |  | 0.24 | 0.24 |
| *Synergistes* | 0 |  | 0 |  | 0 |  | 0.04 | 0.04 |
| *Tenericutes* | 0 |  | 0 |  | 0 |  | 0.04 | 0.04 |
| TM6 | 0.04 | 0.04 | 0.05 | 0.05 | 0.04 | 0.04 | 0.44 | 0.12 |
| TM7 | 0 |  | 0 |  | 0 |  | 0 |  |
| Unclassified | 5.30 | 4.19 | 5.56 | 1.18 | 3.96 | 2.85 | 6.33 | 3.88 |
| *Verrucomicrobia* | 4.0 | 1.15 | 4.91 | 2.18 | 1.98 | 1.98 | 0.20 | 0.12 |
| WPS2 | 0 |  | 0 |  | 0 |  | 0 |  |
| WS2 | 0 |  | 0 |  | 0 |  | 0 |  |
| WS3 | 0 |  | 0 |  | 0 |  | 0 |  |
| ZB3 | 0 |  | 0 |  | 0 |  | 0 |  |

SOM Table 1: Relative abundance of each Bacterial Phyla / Class in *Acropora millepora* (Am) in each experimental treatment; Mean ± Standard Error.

SOM Table 2: Relative abundance of each Bacterial Phyla / Class in *Seriatopora hystrix* (Sh) in each experimental treatment; Mean ± Standard Error.

| **Bacterial Taxa** | **Sh 28°C pH 8.1**  **Mean ± SE** | | **Sh 28°C pH 7.9**  **Mean ± SE** | | **Sh 31°C pH 8.1**  **Mean ± SE** | | **Sh 31°C pH 7.9**  **Mean ± SE** | |
| --- | --- | --- | --- | --- | --- | --- | --- | --- |
| *Acidobacteria* | 0.69 | 0.12 | 0.81 | 0.29 | 0.85 | 0.12 | 0.35 | 0.12 |
| *Actinobacteria* | 0.73 | 0.49 | 1.71 | 0.33 | 2.11 | 0.98 | 1.76 | 0.85 |
| *Bacteroidetes* | 22.24 | 1.99 | 16.26 | 4.67 | 20.49 | 2.20 | 17.07 | 2.69 |
| *Chlamydiae* | 0 |  | 0 |  | 0 |  | 0 |  |
| *Chlorobi* | 0.04 | 0.04 | 0 |  | 0.08 | 0.08 | 0 |  |
| *Chloroflexi* | 0.57 | 0.24 | 0.79 | 0.15 | 1.46 | 1.22 | 0.38 | 0.15 |
| *Cyanobacteria* | 1.22 | 1.06 | 2.74 | 0.92 | 3.70 | 3.13 | 1.63 | 0.80 |
| *Elusimicrobia* | 0.20 | 0.20 | 0 |  | 0 |  | 0 |  |
| *Fibrobacteres* | 0 |  | 0 |  | 0 |  | 0 |  |
| *Firmicutes* | 0.04 | 0.04 | 0.54 | 0.30 | 1.22 | 0.57 | 2.41 | 1.37 |
| *Fusobacteria* | 0 |  | 0 |  | 0 |  | 0 |  |
| *Gemmatimonadetes* | 0.16 | 0.16 | 0.03 | 0.03 | 0.33 | 0 | 0.08 | 0.05 |
| GN02 | 0 |  | 0.03 | 0.03 | 0 |  | 0.03 | 0.03 |
| *Lentisphaerae* | 0.04 | 0.04 | 0.03 | 0.03 | 0 |  | 0.03 | 0.03 |
| *Nitrospirae* | 0 |  | 0.03 | 0.03 | 0 |  | 0 |  |
| NKB19 | 0 |  | 0.03 | 0.03 | 0 |  | 0.03 | 0.03 |
| OD1 | 0 |  | 0 |  | 0 |  | 0 |  |
| PAUC34f | 0 |  | 0 |  | 0.04 | 0.04 | 0 |  |
| *Planctomycetes* | 3.17 | 0.57 | 2.82 | 0.63 | 2.40 | 0.04 | 2.66 | 0.97 |
| *Alphaproteobacteria* | 37.36 | 8.82 | 25.47 | 4.26 | 22.32 | 0.93 | 27.13 | 8.53 |
| *Betaproteobacteria* | 0.16 | 0.08 | 0.11 | 0.05 | 0.16 | 0 | 0.05 | 0.05 |
| *Deltaproteobacteria* | 2.80 | 0.61 | 1.76 | 1.02 | 2.97 | 0.45 | 2.79 | 1.04 |
| *Epsilonproteobacteria* | 0.04 | 0.04 | 0.14 | 0.07 | 8.50 | 8.50 | 5.91 | 5.83 |
| *Gammaproteobacteria* | 12.36 | 3.98 | 25.61 | 6.55 | 14.19 | 0.77 | 11.27 | 2.98 |
| Unclass. *Proteobacteria* | 4.47 | 0 | 9.70 | 7.80 | 3.78 | 0.04 | 3.01 | 0.42 |
| SAR406 | 0 |  | 0 |  | 0 |  | 0 |  |
| SBR1093 | 0.73 | 0.65 | 1.82 | 0.60 | 1.75 | 1.10 | 1.27 | 0.65 |
| *Spirochaetes* | 0.20 | 0.12 | 0 |  | 0.28 | 0.28 | 0.46 | 0.42 |
| *Synergistes* | 0 |  | 0 |  | 0 |  | 0 |  |
| *Tenericutes* | 0 |  | 0 |  | 0 |  | 0 |  |
| TM6 | 0 |  | 0.22 | 0.10 | 0.04 | 0.04 | 0.19 | 0.15 |
| TM7 | 0 |  | 0 |  | 0 |  | 0 |  |
| Unclassified | 11.95 | 1.87 | 8.40 | 1.39 | 12.97 | 0.37 | 21.0 | 10.84 |
| *Verrucomicrobia* | 0.69 | 0.28 | 0.98 | 0.45 | 0.24 | 0.16 | 0.49 | 0.41 |
| WPS2 | 0.04 | 0.04 | 0 |  | 0 |  | 0 |  |
| WS2 | 0 |  | 0 |  | 0 |  | 0 |  |
| WS3 | 0.04 | 0.04 | 0 |  | 0.12 | 0.04 | 0 |  |
| ZB3 | 0.04 | 0.04 | 0 |  | 0 |  | 0 |  |

SOM Table 3: Relative abundance of each Bacterial Phyla / Class in *Heterostegina depressa* (Hd) in each experimental treatment; Mean ± Standard Error.

| **Bacterial Taxa** | **Hd 28°C pH 8.1**  **Mean ± SE** | | **Hd 28°C pH 7.9**  **Mean ± SE** | | **Hd 31°C pH 8.1**  **Mean ± SE** | | **Hd 31°C pH 7.9**  **Mean ± SE** | |
| --- | --- | --- | --- | --- | --- | --- | --- | --- |
| *Acidobacteria* | 1.89 | 1.14 | 0.51 | 0.51 | 0.38 | 0.38 | 0.76 | 0 |
| *Actinobacteria* | 14.77 | 7.95 | 13.64 | 4.01 | 7.95 | 4.92 | 6.57 | 1.01 |
| *Bacteroidetes* | 8.71 | 3.41 | 15.40 | 2.85 | 17.42 | 0 | 11.87 | 1.41 |
| *Chlamydiae* | 0 |  | 0 |  | 0 |  | 0 |  |
| *Chlorobi* | 0 |  | 0 |  | 0 |  | 0 |  |
| *Chloroflexi* | 5.68 | 4.17 | 2.27 | 1.16 | 6.82 | 3.79 | 1.77 | 1.41 |
| *Cyanobacteria* | 0 |  | 0.25 | 0.25 | 0 |  | 0.25 | 0.25 |
| *Elusimicrobia* | 0 |  | 0 |  | 0 |  | 0 |  |
| *Fibrobacteres* | 0 |  | 0 |  | 0 |  | 0 |  |
| *Firmicutes* | 0 |  | 0 |  | 0 |  | 0 |  |
| *Fusobacteria* | 0 |  | 0 |  | 0 |  | 0 |  |
| *Gemmatimonadetes* | 0 |  | 0 |  | 0 |  | 0.51 | 0.51 |
| GN02 | 0 |  | 0 |  | 0 |  | 0 |  |
| *Lentisphaerae* | 0 |  | 0 |  | 0 |  | 0.25 | 0.25 |
| *Nitrospirae* | 0 |  | 0 |  | 0 |  | 0 |  |
| NKB19 | 0 |  | 0 |  | 0 |  | 0 |  |
| OD1 | 0 |  | 0 |  | 0 |  | 0 |  |
| PAUC34f | 0 |  | 0 |  | 0 |  | 0 |  |
| *Planctomycetes* | 1.52 | 0.76 | 3.03 | 2.27 | 1.89 | 1.89 | 2.02 | 0.25 |
| *Alphaproteobacteria* | 21.59 | 2.65 | 20.96 | 3.28 | 20.08 | 1.89 | 24.49 | 3.10 |
| *Betaproteobacteria* | 0 |  | 0.25 | 0.25 | 0 |  | 0.25 | 0.25 |
| *Deltaproteobacteria* | 1.52 | 0.76 | 2.27 | 1.58 | 5.30 | 1.52 | 2.27 | 1.16 |
| *Epsilonproteobacteria* | 0 |  | 0 |  | 0 |  | 0 |  |
| *Gammaproteobacteria* | 16.29 | 2.65 | 19.19 | 2.49 | 14.77 | 4.92 | 30.81 | 1.34 |
| Unclass. *Proteobacteria* | 8.71 | 3.41 | 4.29 | 1.01 | 6.44 | 0.38 | 6.06 | 1.16 |
| SAR406 | 0 |  | 0 |  | 0 |  | 0 |  |
| SBR1093 | 0.76 | 0.76 | 0.51 | 0.51 | 2.65 | 1.89 | 1.52 | 0.87 |
| *Spirochaetes* | 0 |  | 0 |  | 0 |  | 0.25 | 0.25 |
| *Synergistes* | 0 |  | 0 |  | 0 |  | 0 |  |
| *Tenericutes* | 0 |  | 0 |  | 0 |  | 0 |  |
| TM6 | 0 |  | 0 |  | 0.38 | 0.38 | 0 |  |
| TM7 | 0.38 | 0.38 | 0 |  | 0 |  | 0 |  |
| Unclassified | 17.80 | 7.95 | 16.16 | 0.91 | 15.15 | 3.03 | 10.10 | 1.10 |
| *Verrucomicrobia* | 0.38 | 0.38 | 1.26 | 0.51 | 0.38 | 0.38 | 0 |  |
| WPS2 | 0 |  | 0 |  | 0 |  | 0 |  |
| WS2 | 0 |  | 0 |  | 0 |  | 0 |  |
| WS3 | 0 |  | 0 |  | 0.38 | 0.38 | 0.25 | 0.25 |
| ZB3 | 0 |  | 0 |  | 0 |  | 0 |  |

SOM Table 4: Relative abundance of each Bacterial Phyla / Class in *Marginopora vertebralis* (Mv) in each experimental treatment; Mean ± Standard Error.

| **Bacterial Taxa** | **Mv 28°C pH 8.1**  **Mean ± SE** | | **Mv 28°C pH 7.9**  **Mean ± SE** | | **Mv 31°C pH 8.1**  **Mean ± SE** | | **Mv 31°C pH 7.9**  **Mean ± SE** | |
| --- | --- | --- | --- | --- | --- | --- | --- | --- |
| *Acidobacteria* | 0.96 | 0.44 | 1.06 | 0.44 | 1.50 | 0.88 | 1.38 | 0.33 |
| *Actinobacteria* | 1.79 | 0.65 | 1.94 | 0.06 | 1.31 | 0.06 | 2.17 | 0.58 |
| *Bacteroidetes* | 18.83 | 3.82 | 18.75 | 1.88 | 19.81 | 1.06 | 14.54 | 2.04 |
| *Chlamydiae* | 0 |  | 0 |  | 0 |  | 0 |  |
| *Chlorobi* | 0 |  | 0 |  | 0 |  | 0.04 | 0.04 |
| *Chloroflexi* | 2.50 | 1.00 | 3.06 | 0.69 | 2.69 | 1.31 | 2.71 | 0.63 |
| *Cyanobacteria* | 0.63 | 0.33 | 1.44 | 0.19 | 1.81 | 0.19 | 0.42 | 0.11 |
| *Elusimicrobia* | 0 |  | 0 |  | 0 |  | 0 |  |
| *Fibrobacteres* | 0 |  | 0.13 | 0.13 | 0 |  | 0 |  |
| *Firmicutes* | 0.08 | 0.08 | 0 |  | 0.06 | 0.06 | 0 |  |
| *Fusobacteria* | 0.04 | 0.04 | 0 |  | 0 |  | 0 |  |
| *Gemmatimonadetes* | 0.08 | 0.04 | 0 |  | 0 |  | 0.08 | 0.08 |
| GN02 | 0 |  | 0.06 | 0.06 | 0.38 | 0.13 | 0.29 | 0.23 |
| *Lentisphaerae* | 0.04 | 0.04 | 1.19 | 0.81 | 0 |  | 0.17 | 0.08 |
| *Nitrospirae* | 0 |  | 0.13 | 0.13 | 0 |  | 0 |  |
| NKB19 | 0 |  | 0 |  | 0 |  | 0 |  |
| OD1 | 0.04 | 0.04 | 0 |  | 0 |  | 0 |  |
| PAUC34f | 0 |  | 0 |  | 0 |  | 0 |  |
| *Planctomycetes* | 4.25 | 1.94 | 3.81 | 0.94 | 8.88 | 3.50 | 5.38 | 2.04 |
| *Alphaproteobacteria* | 34.54 | 13.63 | 23.06 | 9.19 | 17.50 | 2.38 | 26.33 | 2.38 |
| *Betaproteobacteria* | 1.00 | 0.38 | 0.56 | 0.31 | 0.19 | 0.06 | 1.29 | 0.74 |
| *Deltaproteobacteria* | 1.25 | 0.31 | 3.00 | 0.13 | 5.06 | 0.31 | 2.79 | 0.71 |
| *Epsilonproteobacteria* | 0 |  | 0 |  | 0 |  | 0 |  |
| *Gammaproteobacteria* | 11.71 | 1.61 | 14.75 | 2.00 | 19.63 | 2.00 | 21.33 | 4.14 |
| Unclass. *Proteobacteria* | 2.46 | 0.48 | 7.38 | 4.25 | 2.88 | 0 | 4.75 | 0.94 |
| SAR406 | 0 |  | 0 |  | 0 |  | 0 |  |
| SBR1093 | 0.46 | 0.18 | 1.00 | 0.38 | 2.44 | 1.06 | 0.88 | 0.07 |
| *Spirochaetes* | 0.08 | 0.04 | 0 |  | 0.13 | 0.13 | 0.08 | 0.08 |
| *Synergistes* | 0 |  | 0 |  | 0 |  | 0 |  |
| *Tenericutes* | 0 |  | 0 |  | 0 |  | 0 |  |
| TM6 | 0.21 | 0.21 | 0.13 | 0 | 0.13 | 0 | 0.29 | 0.04 |
| TM7 | 0 |  | 0 |  | 0 |  | 0 |  |
| Unclassified | 18.38 | 4.94 | 16.50 | 1.88 | 14.94 | 0.94 | 13.50 | 1.50 |
| *Verrucomicrobia* | 0.58 | 0.08 | 2.00 | 0.25 | 0.56 | 0.06 | 1.25 | 0.52 |
| WPS2 | 0 |  | 0 |  | 0 |  | 0 |  |
| WS2 | 0.04 | 0.04 | 0 |  | 0 |  | 0 |  |
| WS3 | 0.04 | 0.04 | 0.06 | 0.06 | 0.13 | 0 | 0.33 | 0.11 |
| ZB3 | 0 |  | 0 |  | 0 |  | 0 |  |

SOM Table 5: Relative abundance of each Bacterial Phyla / Class in *Hydrolithon onkodes* (Ho) in each experimental treatment; Mean ± Standard Error.

| **Bacterial Taxa** | **Ho 28°C pH 8.1**  **Mean ± SE** | | **Ho 28°C pH 7.9**  **Mean ± SE** | | **Ho 31°C pH 8.1**  **Mean ± SE** | | **Ho 31°C pH 7.9**  **Mean ± SE** | |
| --- | --- | --- | --- | --- | --- | --- | --- | --- |
| *Acidobacteria* | 0.20 | 0.11 | 0.20 | 0.11 | 0.88 | 0.46 | 0.32 | 0.17 |
| *Actinobacteria* | 0.36 | 0.12 | 0.40 | 0.11 | 0.48 | 0 | 0.28 | 0.22 |
| *Bacteroidetes* | 26.32 | 1.59 | 30.47 | 2.44 | 24.77 | 3.87 | 34.01 | 2.31 |
| *Chlamydiae* | 0 |  | 0 |  | 0.04 | 0.04 | 0 |  |
| *Chlorobi* | 0 |  | 0.12 | 0.12 | 0 |  | 0.04 | 0.04 |
| *Chloroflexi* | 0.60 | 0 | 0.04 | 0.04 | 0.40 | 0.08 | 0.36 | 0.07 |
| *Cyanobacteria* | 2.67 | 1.18 | 2.67 | 1.05 | 3.39 | 0.42 | 2.23 | 0.17 |
| *Elusimicrobia* | 0 |  | 0 |  | 0 |  | 0 |  |
| *Fibrobacteres* | 0 |  | 0 |  | 0 |  | 0 |  |
| *Firmicutes* | 0 |  | 0 |  | 0 |  | 0.04 | 0.04 |
| *Fusobacteria* | 0.04 | 0.04 | 0 |  | 0 |  | 0 |  |
| *Gemmatimonadetes* | 0 |  | 0 |  | 0 |  | 0 |  |
| GN02 | 0.08 | 0.08 | 0.04 | 0.04 | 0 |  | 0.04 | 0.04 |
| *Lentisphaerae* | 0 |  | 0.16 | 0.11 | 0.04 | 0.04 | 0.08 | 0.08 |
| *Nitrospirae* | 0 |  | 0 |  | 0 |  | 0 |  |
| NKB19 | 0 |  | 0 |  | 0 |  | 0 |  |
| OD1 | 0 |  | 0 |  | 0.04 | 0.04 | 0 |  |
| PAUC34f | 0 |  | 0 |  | 0 |  | 0 |  |
| *Planctomycetes* | 4.82 | 0.21 | 3.94 | 1.48 | 3.66 | 1.10 | 2.71 | 1.01 |
| *Alphaproteobacteria* | 26.09 | 3.15 | 24.93 | 0.74 | 37.00 | 1.82 | 25.89 | 2.67 |
| *Betaproteobacteria* | 0.16 | 0.04 | 0.16 | 0.08 | 0 |  | 0.04 | 0.04 |
| *Deltaproteobacteria* | 0.84 | 0.07 | 0.76 | 0.29 | 1.19 | 0.07 | 0.84 | 0.37 |
| *Epsilonproteobacteria* | 0 |  | 0.04 | 0.04 | 0.04 | 0.04 | 0 |  |
| *Gammaproteobacteria* | 20.87 | 3.09 | 19.51 | 1.96 | 15.61 | 3.42 | 21.35 | 2.68 |
| Unclass. *Proteobacteria* | 7.77 | 2.04 | 9.12 | 0.34 | 3.74 | 0.86 | 3.31 | 0.74 |
| SAR406 | 0 |  | 0 |  | 0 |  | 0 |  |
| SBR1093 | 0.08 | 0.04 | 0 |  | 0 |  | 0.20 | 0.04 |
| *Spirochaetes* | 0.48 | 0.28 | 1.12 | 0.46 | 1.51 | 0.59 | 0.96 | 0.32 |
| *Synergistes* | 0 |  | 0 |  | 0 |  | 0 |  |
| *Tenericutes* | 0 |  | 0 |  | 0 |  | 0 |  |
| TM6 | 0 |  | 0 |  | 0 |  | 0 |  |
| TM7 | 0 |  | 0 |  | 0 |  | 0 |  |
| Unclassified | 6.93 | 0.86 | 5.97 | 0.38 | 6.77 | 1.58 | 6.69 | 1.28 |
| *Verrucomicrobia* | 1.71 | 1.30 | 0.36 | 0.07 | 0.44 | 0.11 | 0.64 | 0.33 |
| WPS2 | 0 |  | 0 |  | 0 |  | 0 |  |
| WS2 | 0 |  | 0 |  | 0 |  | 0 |  |
| WS3 | 0 |  | 0 |  | 0 |  | 0 |  |
| ZB3 | 0 |  | 0 |  | 0 |  | 0 |  |

SOM Table 6: Relative abundance of each Bacterial Phyla / Class in *Echinometra* sp. A (Ec) in each experimental treatment; Mean ± Standard Error.

| **Bacterial Taxa** | **Ec 28°C pH 8.1**  **Mean ± SE** | | **Ec 28°C pH 7.9**  **Mean ± SE** | | **Ec 31°C pH 8.1**  **Mean ± SE** | | **Ec 31°C pH 7.9**  **Mean ± SE** | |
| --- | --- | --- | --- | --- | --- | --- | --- | --- |
| *Acidobacteria* | 0.28 | 0.14 | 0.04 | 0.0 | 0.02 | 0.02 | 0.07 | 0.05 |
| *Actinobacteria* | 1.76 | 0.93 | 0.13 | 0.10 | 0.22 | 0.08 | 5.59 | 3.88 |
| *Bacteroidetes* | 17.25 | 9.43 | 13.40 | 8.36 | 4.82 | 1.40 | 11.46 | 3.72 |
| *Chlamydiae* | 0 |  | 0 |  | 0 |  | 0 |  |
| *Chlorobi* | 0 |  | 0.70 | 0.70 | 0 |  | 0.75 | 0.41 |
| *Chloroflexi* | 0.35 | 0.35 | 0 |  | 0 |  | 0.24 | 0.24 |
| *Cyanobacteria* | 0.44 | 0.29 | 0.20 | 0.20 | 0.24 | 0.08 | 1.32 | 1.32 |
| *Elusimicrobia* | 0 |  | 0 |  | 0 |  | 0 |  |
| *Fibrobacteres* | 0.04 | 0.04 | 0.02 | 0.02 | 0 |  | 1.05 | 1.05 |
| *Firmicutes* | 1.61 | 0.55 | 0.44 | 0.36 | 0.17 | 0.03 | 6.11 | 4.01 |
| *Fusobacteria* | 1.27 | 0.95 | 1.17 | 0.84 | 0.51 | 0.04 | 3.26 | 2.46 |
| *Gemmatimonadetes* | 0 |  | 0 |  | 0 |  | 0 |  |
| GN02 | 0.02 | 0.02 | 0 |  | 0 |  | 0 |  |
| *Lentisphaerae* | 0.11 | 0.11 | 0 |  | 0.07 | 0.05 | 0 |  |
| *Nitrospirae* | 0 |  | 0 |  | 0 |  | 0 |  |
| NKB19 | 0 |  | 0 |  | 0 |  | 0 |  |
| OD1 | 0 |  | 0 |  | 0 |  | 0.02 | 0.02 |
| PAUC34f | 0 |  | 0 |  | 0 |  | 0 |  |
| *Planctomycetes* | 0.68 | 0.36 | 0.66 | 0.33 | 0.17 | 0.11 | 0.53 | 0.53 |
| *Alphaproteobacteria* | 23.52 | 7.90 | 9.48 | 1.24 | 50.79 | 23.15 | 11.92 | 4.68 |
| *Betaproteobacteria* | 1.30 | 0.98 | 0.90 | 0.71 | 0.06 | 0.03 | 0.94 | 0.43 |
| *Deltaproteobacteria* | 2.26 | 0.66 | 4.84 | 3.56 | 0.37 | 0.28 | 2.40 | 1.85 |
| *Epsilonproteobacteria* | 0.02 | 0.02 | 0 |  | 0 |  | 0 |  |
| *Gammaproteobacteria* | 37.88 | 15.49 | 58.56 | 14.42 | 27.56 | 12.38 | 38.91 | 2.68 |
| Unclass. *Proteobacteria* | 0.94 | 0.66 | 0.94 | 0.62 | 0.17 | 0.11 | 1.78 | 0.83 |
| SAR406 | 0.02 | 0.02 | 0 |  | 0.02 | 0.02 | 0 |  |
| SBR1093 | 0.20 | 0.17 | 0 |  | 0.07 | 0.05 | 0.09 | 0.09 |
| *Spirochaetes* | 0.02 | 0.02 | 2.13 | 1.61 | 0.13 | 0.10 | 0.79 | 0.43 |
| *Synergistes* | 0 |  | 0 |  | 0 |  | 0 |  |
| *Tenericutes* | 0 |  | 0.04 | 0.04 | 0 |  | 0 |  |
| TM6 | 0.04 | 0.04 | 0 |  | 0 |  | 0 |  |
| TM7 | 0 |  | 0 |  | 0 |  | 0 |  |
| Unclassified | 9.88 | 1.15 | 6.34 | 2.31 | 14.48 | 11.63 | 12.32 | 4.10 |
| *Verrucomicrobia* | 0.09 | 0.07 | 0.02 | 0.02 | 0.15 | 0.07 | 0.46 | 0.27 |
| WPS2 | 0 |  | 0 |  | 0 |  | 0 |  |
| WS2 | 0 |  | 0 |  | 0 |  | 0 |  |
| WS3 | 0.04 | 0.04 | 0 |  | 0 |  | 0 |  |
| ZB3 | 0 |  | 0 |  | 0 |  | 0 |  |

SOM Table 7: Relative abundance of each Bacterial Phyla / Class in Seawater (Sw) in each experimental treatment; Mean ± Standard Error.

| **Bacterial Taxa** | **Sw 28°C pH 8.1**  **Mean ± SE** | | **Sw 28°C pH 7.9**  **Mean ± SE** | | **Sw 31°C pH 8.1**  **Mean ± SE** | | **Sw 31°C pH 7.9**  **Mean ± SE** | |
| --- | --- | --- | --- | --- | --- | --- | --- | --- |
| *Acidobacteria* | 0 |  | 0.02 | 0.02 | 0.02 | 0.02 | 0.02 | 0.02 |
| *Actinobacteria* | 1.77 | 0.16 | 3.10 | 0.25 | 2.75 | 0.37 | 2.06 | 0.24 |
| *Bacteroidetes* | 8.95 | 1.62 | 9.14 | 0.66 | 10.35 | 1.29 | 8.72 | 0.44 |
| *Chlamydiae* | 0 |  | 0 |  | 0 |  | 0 |  |
| *Chlorobi* | 0 |  | 0 |  | 0 |  | 0 |  |
| *Chloroflexi* | 0.02 | 0.02 | 0.02 | 0.02 | 0.08 | 0.08 | 0 |  |
| *Cyanobacteria* | 12.35 | 2.35 | 12.58 | 1.08 | 11.81 | 0.20 | 17.95 | 0.98 |
| *Elusimicrobia* | 0 |  | 0 |  | 0 |  | 0 |  |
| *Fibrobacteres* | 0 |  | 0 |  | 0.02 | 0.02 | 0 |  |
| *Firmicutes* | 0 |  | 0.02 | 0.02 | 0.06 | 0.04 | 0 |  |
| *Fusobacteria* | 0 |  | 0 |  | 0 |  | 0 |  |
| *Gemmatimonadetes* | 0.02 | 0.02 | 0 |  | 0 |  | 0 |  |
| GN02 | 0.02 | 0.02 | 0.08 | 0.08 | 0.06 | 0.04 | 0 |  |
| *Lentisphaerae* | 0 |  | 0 |  | 0.04 | 0.02 | 0.04 | 0.02 |
| *Nitrospirae* | 0 |  | 0.02 | 0.02 | 0.02 | 0.02 | 0 |  |
| NKB19 | 0 |  | 0 |  | 0 |  | 0.02 | 0.02 |
| OD1 | 0 |  | 0 |  | 0 |  | 0 |  |
| PAUC34f | 0.06 | 0.06 | 0.08 | 0.06 | 0.02 | 0.02 | 0 |  |
| *Planctomycetes* | 0.21 | 0.10 | 0.06 | 0.04 | 0.29 | 0.08 | 0.46 | 0.12 |
| *Alphaproteobacteria* | 60.30 | 3.97 | 60.77 | 2.79 | 55.74 | 2.23 | 55.01 | 2.10 |
| *Betaproteobacteria* | 1.17 | 0.37 | 0.85 | 0.20 | 1.19 | 0.04 | 1.12 | 0.22 |
| *Deltaproteobacteria* | 0.25 | 0.04 | 0.29 | 0.04 | 0.40 | 0.13 | 0.21 | 0.04 |
| *Epsilonproteobacteria* | 0 |  | 0 |  | 0 |  | 0 |  |
| *Gammaproteobacteria* | 7.04 | 0.71 | 5.04 | 0.13 | 8.08 | 0.29 | 6.25 | 0.59 |
| Unclass. *Proteobacteria* | 0.85 | 0.15 | 0.94 | 0.22 | 1.12 | 0.10 | 1.06 | 0.13 |
| SAR406 | 0.77 | 0.31 | 0.92 | 0.25 | 0.96 | 0.08 | 0.44 | 0.12 |
| SBR1093 | 0 |  | 0 |  | 0.08 | 0.02 | 0.04 | 0.02 |
| *Spirochaetes* | 0 |  | 0 |  | 0 |  | 0 |  |
| *Synergistes* | 0 |  | 0 |  | 0 |  | 0 |  |
| *Tenericutes* | 0.02 | 0.02 | 0.02 | 0.02 | 0.04 | 0.04 | 0.02 | 0.02 |
| TM6 | 0.02 | 0.02 | 0 |  | 0.04 | 0.04 | 0.04 | 0.04 |
| TM7 | 0 |  | 0 |  | 0 |  | 0 |  |
| Unclassified | 2.39 | 0.68 | 2.58 | 0.40 | 3.37 | 0.37 | 3.16 | 0.06 |
| *Verrucomicrobia* | 3.75 | 0.56 | 3.44 | 0.40 | 3.44 | 0.27 | 3.37 | 0.06 |
| WPS2 | 0 |  | 0 |  | 0 |  | 0 |  |
| WS2 | 0 |  | 0 |  | 0 |  | 0 |  |
| WS3 | 0 |  | 0 |  | 0 |  | 0 |  |
| ZB3 | 0.04 | 0.02 | 0.02 | 0.02 | 0.02 | 0.02 | 0 |  |
